# Pollinizer’ Effects on Olive Seed Set, Size and Abortion

**DOI:** 10.3390/plants15050813

**Published:** 2026-03-06

**Authors:** Julián Cuevas, Fernando M. Chiamolera, Alenka Baruca Arbeiter, Marina Raboteg Božiković, Gabriela Vuletin Selak

**Affiliations:** 1 Department of Agronomy, University of Almería, ceiA3, 04120 Almería, Spain; fmc1984@ual.es; 2 Faculty of Mathematics, Natural Sciences and Information Technologies, University of Primorska, 6000 Koper, Slovenia; alenka.arbeiter@upr.si; 3 Institute for Adriatic Crops and Karst Reclamation, 21000 Split, Croatia; marina.raboteg@krs.hr (M.R.B.); gabriela.vuletin.selak@krs.hr (G.V.S.); 4 Centre of Excellence for Biodiversity and Molecular Plant Breeding, 10000 Zagreb, Croatia

**Keywords:** *Olea europaea*, cross-pollination, paternal and maternal effects on seeds, twin seeds, seed abortion, self-incompatibility

## Abstract

Olive is a self-incompatible species that usually produces one-seeded drupes, although double-seeded fruits and seedless fruit due to seed abortion may occur. This study evaluated the effects of different pollinizers on seeding pattern in three self-incompatible cultivars: ‘Arbosana’, ‘Koroneiki’ and ‘Sikitita’. Fruit and seed set, seed abortion, double seeding and seed weight were analyzed. Maternal effects were confirmed with significant differences between cultivars. ‘Sikitita’ produced a higher-than-expected proportion of double-seeded fruit, whereas ‘Arbosana’ and ‘Koroneiki’ produced fewer. In contrast, ‘Koroneiki’ showed a higher incidence of empty endocarps, while ‘Sikitita’ produced fewer. Paternal effects on seeding pattern were generally not significant, although marginal differences were observed in ‘Arbosana’ and ‘Koroneiki’ depending on pollination treatment. Some pollination crosses were more successful, with ‘Sikitita’ pollen fathering most seeds in ‘Koroneiki’ and ‘Arbosana’, with reciprocal success for ‘Arbosana’ pollen in ‘Sikitita’. A striking discovery was that different fathers often sired twin seeds within one single fruit. Finally, seed number affected fruit development. Thus, total seed and endocarp mass increased as viable seed number did. Fruits with aborted seeds had smaller stones, whereas stones enclosing two seeds were heavier. Seed, endocarp, pulp and fruit weights were positively correlated across cultivars and pollination conditions.

## 1. Introduction

Olive (*Olea europaea* L.) is an extremely important fruit crop in the Mediterranean basin, as well as now elsewhere in the world. Cultivated since ancient times on both sides of the Mediterranean Sea, north and south, the olive occupies an area of 11.1 million ha worldwide, producing an average tonnage of 20.3 Mt per year. The olive is propagated vegetatively, mainly by cuttings [1]. Therefore, cultivars are genetically homogeneous and monovarietal orchards are the norm. However, olive is self-incompatible. This condition has been proven in the species as a whole [2], and repeatedly in the most important cultivars, among them those that make up very large super-high-density orchards [3,4,5]. This apparent paradox is explained because olive is wind-pollinated and its pollen might travel great distances [6], making cross-pollination possible from distant plants of different cultivars [7]. The situation can be riskier, however, in new areas of olive cultivation, where no other cultivars grow nearby, and when very large super-high-density monovarietal orchards are designed.

The preferential allogamy in olive is based on an incompatibility reaction on the stigma of the recipient cultivar. Thus, when self-pollination occurs, most, if not all, pollen grains, are blocked soon after germination, allowing very few, if any, pollen tubes to grow in the style toward the ovary. In contrast, in cross-pollination, the stigma does not reject the pollen grains and dozens of pollen tubes grow rapidly through the transmitting tissue of the style achieving fertilization [8]. With very few exceptions, fertilization takes place only in one of the four ovules enclosed in the ovary, since generally only one pollen tube accesses the ovary [9]. The olive fruit is therefore a one-seeded drupe, although, on some occasions, double-seeded fruit and fruit with aborted seeds are formed and found at harvest [10].

The self-incompatibility reactions described above reflect a preference for cross-pollination and delay and low levels of fertilization when only self-pollinating grains reach the stigma. This self-incompatibility has been recently attributed to a sporophytic self-incompatibility (SSI) system. Several research groups have proposed two or more incompatibility groups, although this theory clearly needs some refinement [11,12].

Although olive self-incompatibility is no longer questioned, the ultimate effects of the strong selection of the winner pollen tube on its offspring have not yet been explored. Since the self-incompatibility reaction results in the selection of the fittest pollen grain genotype for achieving ovule fertilization, it is unknown what effects different pollinizers may have on the fate of the seeds produced. We aim to take advantage of an extensive recent study in which we determined the self-incompatibility response and the effects of cross-pollination in three low-vigor cultivars frequently used in super-high-density orchards [3,4,5].

Taking this information into consideration, the objective of this work was to determine the effects of different pollinizers on seeding patterns, reporting the influence of different pollen donors on seed set, seed abortion, double seed formation and seed size in ‘Arbosana’, ‘Sikitita’ and ‘Koroneiki’ olive cultivars.

## 2. Results

All three cultivars behaved as strongly self-incompatible, producing a very small amount of fruit under self-pollination treatment. Final fruit set was significantly lower for self-pollination compared to open-pollination in all cultivars. Fruit set under self-pollination was also significantly reduced compared to most cross-pollination treatments, but not in the case of ‘Koroneiki’ and ‘Sikitita’ when crossed with pollen of ‘Arbequina’, illustrating that these cultivars are mutually inter-incompatible with the latter (Table 1). The results of the crossings seldom affected the weight of the fruit, although in all cases open-pollination slightly enhanced fruit size with respect to self-pollination (Table 1).

The seed paternity assigned to the different pollinizers in the open-pollinated flowers did not exactly match the compatibility shown by the fruit set response. However, no single seed from open-pollination was the result of self-fertilization in any of the recipient cultivars (‘Arbosana’, ‘Koroneiki’ or ‘Sikitita’), confirming their self-incompatibility. Some reciprocal preferences revealed by the fruit set results were also observed in the mates. Specifically, in the fruits of ‘Sikitita’, most of the seeds were produced by the pollen of ‘Arbosana’, while in the open-pollinated flowers of ‘Arbosana’, most of the seeds were produced as a result of fertilization with pollen from ‘Sikitita’, with ‘Arbequina’ close (Table 2). In open-pollinated flowers of ‘Koroneiki’, most seeds were produced by ‘Sikitita’ pollen, with ‘Arbosana’ following, but ‘Koroneiki’ pollen failed to father many seeds in ‘Sikitita’ (Table 2), despite the fruit set suggesting high compatibility between them (Table 1).

Some variations in paternity achieved by the different pollinizers were found among trees of the same recipient cultivars, suggesting some positional effects of the trees in the row. In this regard, Chi-square tests did not reach statistical significance for the pattern of paternity depending on tree replication for ‘Arbosana’ or ‘Sikitita’ (*p* = 0.218 and 0.127). However, the pattern of fathering pattern was statistically different in ‘Koroneiki’ plants (*p* = 0.030), mainly due to the irregular paternity achieved in some trees by the pollen from ‘Sikitita’ and ‘Arbosana’, but also from ‘Tosca’, while ‘Arbequina’ pollen fathered a similar number of seeds in the different trees of ‘Koroneiki’.

Maternal effects on seeding pattern, regardless of pollination treatment, were confirmed. Chi-square analysis showed significant differences among cultivars in the proportion of empty endocarps and in the proportion of endocarps containing one or two healthy seeds (overall Chi-square = 22.97; *p* = 0.0001). In this regard, ‘Sikitita’ produced endocarps with two seeds more often than expected, while ‘Arbosana’ and ‘Koroneiki’ produced very few, confirming a varietal effect on this anomaly. In contrast, ‘Koroneiki’ produced empty endocarps due to seed abortion more often than expected, while ‘Sikitita’ produced fewer than expected.

To determine in which measure the pollinizers affected the seeding pattern and seed characteristics, the results are presented below for each maternal cultivar individually.

### 2.1. ‘Arbosana’

Pollination treatments did not significantly (*p* = 0.096) modify the pattern of seed set in ‘Arbosana’ fruit, although open-pollination and cross-pollination with ‘Koroneiki’ produced fewer fruit with empty endocarps, while self-pollination and pollination with ‘Arbequina’ produced slightly more. Double seeds were observed only in three fruits of ‘Arbosana’, one from open-pollination, the second from pollination with ‘Sikitita’ and the third from self-pollination. The twin seeds found in the self-pollination treatment were both dead at harvest. It is remarkable that the twin seeds found inside the endocarp in the open-pollination treatment were fathered by different cultivars: one seed was the result of fertilization by ‘Sikitita’ pollen, and the second by ‘Arbequina’. No significant effects of pollination treatments were found on the weight of healthy single seeds (*p* = 0.16) or on total seed tissues (including in the analysis decaying and double seeds) (*p* = 0.40). The effect of pollination treatments on endocarp weight was low and not significant either (*p* = 0.24).

When analyzing the DNA of ‘Arbosana’ seeds collected from open-pollination treatment (*n* = 80; 76 with DNA extracted), we found that no seed was the result of self-fertilization (Table 2). In contrast, all seeds were the product of cross-pollination and included seeds fathered by all cultivars present in the experimental orchard. Nonetheless, some pollinizers fathered more seeds than others did. Specifically, 37 out of 76 fruits included a seed fathered by ‘Sikitita’ (one fruit with twin seeds), while 27 seeds were fathered by pollen of ‘Arbequina’ (Table 2). ‘Sikitita’ and ‘Arbequina’ pollen produced significantly more seeds than ‘Tosca’ and ‘Koroneiki’ pollen (*p* < 0.001).

In addition to the successful paternity producing more seeds, the seeds fathered by ‘Sikitita’ in open-pollinated flowers were among the heaviest (41.2 mg), although they were not significantly heavier than the seeds produced by other pollen donors (Table 3) (*p* = 0.24; Student’s t-test for ‘Sikitita’-pollen-sired seeds versus ‘Koroneiki’-sired seeds). Furthermore, the range of weight for seeds fathered by the different pollen donors overlapped. Nonetheless, pollinizers fathering a higher number of seeds (‘Sikitita’ and ‘Arbequina’) were able to make them a little bit heavier, while ‘Koroneiki’ pollen not only fathered a lower number of seeds but also produced the lightest seeds.

The number of seeds per fruit had a clearer effect on seed weight (Table 4). The average weight of each seed coming from the endocarps containing two healthy seeds (only two fruits) was less than the average weight of a single seed. However, the total weight of both seeds was much higher than the average weight of a single seed and than the weight of any single seed (Table 4). As expected, dead seeds weighed significantly (*p* < 0.0001) less than single healthy seeds. The weight of the decaying seed was, however, highly variable, with values sometimes close to that of viable seeds. The effect of the number of seeds per fruit on endocarp size was lower and not significant (180.1 versus 189.8 mg for stones with aborted seeds versus stones with healthy seeds; *p* = 0.90). Finally, the very few endocarps containing two seeds were slightly heavier on average than the standard one-seed endocarps, not only because the total weight of the two seeds enclosed was higher, but also because double seeding promoted the development of a larger stone (211.3 mg).

The correlation and regression analyses carried out on the total number of fruits of ‘Arbosana’ showed a strong relationship between the weight of single healthy seeds and the weight of the endocarp (maternal tissue) enclosing them (*r* = 0.712; *p* < 0.0001). The linear equation of endocarp weight = 2.66 × seed weight + 0.08 (in grams) described well such a highly significant relationship (*r*^2^ = 0.51; *p* < 0.0001). Finally, data from the small sample of fruits harvested to determine parameters defining fresh fruit weight also proved a significant relationship between seed weight and fruit weight in this cultivar. The coefficient of correlation between these two parameters was *r* = 0.49 with a *p* = 0.02. Removing from the correlation analysis the data of the four fruits with empty endocarps improved the results (*r* = 0.65; *p* = 0.006), indicating the variable effect of seed abortion. The following linear equation described well the dependence of fruit weight on seed weight (independently if they were alive or dead): fruit weight = 10.22 × seed weight + 1.00 (in grams) (*r*^2^ = 0.24; *p* = 0.028). The same results can be applied to the weight of the pulp (exocarp and mesocarp), indicating the strong influence of the size of the seed on the size of the tissues synthesizing most of the olive oil (*r* = 0.44; *p* = 0.05). A larger and very significant relationship was found between fruit and endocarp (maternal tissue only) (*r* = 0.94; *p* < 0.0001). No bi-seeded fruits were found in this smaller sample, confirming the low occurrence of this circumstance in ‘Arbosana’.

### 2.2. ‘Koroneiki’

As in ‘Arbosana’, the seeding pattern in ‘Koroneiki’ was only marginally affected by the pollination treatment (overall Chi-square = 13.71; *p* = 0.089), although open-pollination again produced fewer empty endocarps than expected and the only two fruits with double seeds, while self-pollination and cross-pollination with ‘Arbequina’ produced more fruit with aborted seeds than expected. As previously noted, the abortion of the seed regardless of pollination treatments affected its weight. Seed tissues weighed significantly less (*p* < 0.0001) in fruit with decaying seeds than in fruit where the single seed was healthy at harvest (Table 5). As in ‘Arbosana’, the weight of the decaying seed was highly variable, with some aborted seeds similarly sized to healthy seeds (Table 5). Very few, only two, bi-seeded fruit were detected in ‘Koroneiki’. The average weight of each of the twin seeds was clearly diminished compared to the weight of single healthy seeds (Table 5). As noted before in ‘Arbosana’, the effect of seed abortion on the weight of the endocarps was smaller and not significant (174.8 vs. 167.5 mg, for endocarps with and without healthy seeds; *p* = 0.14).

On the other hand, seed weight in single-seeded fruit was again strongly related to the size of the endocarp (*r* = 0.71; *p* < 0.0001). A linear equation explains this highly significant relationship: endocarp weight = 3.97 × seed weight + 0.04 (in grams). The significance of the relationship between these two organs was greatly weakened if in the analysis we include empty endocarps (*r* = 0.17; *p* = 0.05), reflecting the variable influence of decaying seeds on endocarp weight. Only two bi-seeded fruits were found, with the seeds fathered by different genotypes (one fruit with the seeds fathered by ‘Sikitita’ and ‘Arbosana’, and the second fruit with twin seeds the product of fertilization by ‘Sikitita’ and ‘Tosca’).

As was the case with ‘Arbosana’, no significant differences were found in the weight of single seeds of ‘Koroneiki’ (*p* = 0.75) in response to the pollination treatments. Although self-pollination again produced the lightest seeds, they were remarkably similar in all treatments, with the average weight of healthy single seeds being between 32.6 mg (in self-pollination) and 34.4 (under open-pollination). Pollination treatments did not have significant effects on stone size regardless of whether we include or not the empty endocarps and the very few endocarps containing two seeds (*p* = 0.64 and 0.70, respectively).

In fruit produced from open-pollinated flowers of ‘Koroneiki’, most of the seeds were the product of fertilization by ‘Sikitita’ pollen (41 out of 70 seeds from which DNA was successfully extracted). ‘Arbosana’ pollen fathered 16 seeds, one of them in a bi-seeded fruit. ‘Arbequina’ pollen produced nine seeds, while only six were produced by ‘Tosca’ (one as a twin seed) (Table 2). Two fruits of this sample were bi-seeded, and, in both, one seed was fathered by ‘Sikitita’ while the second seed was fathered by ‘Arbequina’ or ‘Tosca’. Again, no seeds were a product of self-fertilization. Statistical analysis confirmed that ‘Sikitita’ produced significantly more seeds than any other pollinizer on ‘Koroneiki’ fruits (*p* < 0.001). Nonetheless, this preference for ‘Sikitita’ pollen was not expressed in the size of the seeds, which once again reached a similar weight in all crosses (Table 3). As before, the range of seed weight overlapped between 22.6 and 45.9 mg. Despite this large variability, the coefficient of variation in each group of seeds was similar.

No bi-seeded fruits were found in the small sample harvested to determine the relationship between seed and fruit weights. In contrast, 20% of the fruit (5 out of 20 fruits) contained empty endocarps. As expected, seed weight was significantly related to fresh fruit weight, with a coefficient of correlation of *r* = 0.46 (*p* = 0.042), although the coefficient of correlation was clearly improved when the fruit with empty endocarps were removed from the analysis (*r* = 0.74; *p* = 0.002), and similarly when correlated only with oily tissues (exo- and mesocarp) (*r* = 0.71; *p* = 0.003). The following linear equation defined fruit weight depending on seed weight, seed dead or alive: fruit weight = 13.02 × seed weight + 1.93 (in grams) (*r*^2^ = 0.21; *p* = 0.042). The relationship between endocarp and the whole fruit weight was even higher (*r* = 0.90; *p* < 0.0001).

### 2.3. ‘Sikitita’

Regarding the seeding pattern, ‘Sikitita’ fruits were quite distinct from the other two cultivars (*p* = 0.0001). In this regard, the percentage of empty endocarps due to late seed abortion was low in ‘Sikitita’, only 5%, but it was especially common in replication (tree) number one under open-pollination treatment, where up to 25% of the fruit had empty endocarps. This tree also produced a high percentage of empty endocarps in fruit coming from pollination with ‘Arbequina’ (37.5%), but this effect was not observed in the other three replicates (trees) where no fruit had empty endocarps. No empty endocarps were the product of pollination with ‘Koroneiki’, while under self-pollination 7% of the fruit contained empty endocarps, therefore affecting the weight of the endocarp as explained before. Contrary to what was observed on ‘Arbosana’ and ‘Koroneiki’, twin seeds were often found in the fruit of ‘Sikitita’, especially under open-pollination, where up to 12.5% of the fruit was double-seeded. This percentage was 10% when applying ‘Koroneiki’ pollen. In only two cases, the second seed was dead. It is worthwhile to comment that in one fruit three viable seeds were found. Pollination with ‘Arbequina’ did not produce twin seeds, but under self-pollination 7% of the fruits were also bi-seeded. Despite the clear distinction of the seeding pattern from the seeding of previous cultivars, no significant effect of pollination treatments (paternal effect) could be proved with it (overall Chi-square = 8.56; *p* = 0.38), and the production of empty endocarps and one- and two-seeded fruits was regularly distributed among the different pollination treatments.

As before, pollination treatments were not able to significantly modify the weight of the seeds enclosed in the endocarp (*p* = 0.16), although cross-pollination with ‘Koroneiki’ produced substantially heavier seeds (52.9 mg versus 44.4 for self-pollination), partly because using this pollen led to the appearance of more double-seeded fruit and even one fruit with three healthy seeds. When we compared only single-seeded fruits, the effect of the pollen source was barely maintained (*p* = 0.32). Similarly, ‘Koroneiki’ pollen produced heavier endocarps, although the differences only reached statistical significance against the fruit produced in cross-pollination with ‘Arbequina’ pollen (340.0 versus 284.0 mg; *p* = 0.03); this effect remains even if we remove from the data set the empty endocarps and those containing two and three seeds (*p* = 0.002).

As deduced from the above comments, the number of seeds in the fruits of ‘Sikitita’ had a strong effect on total plant investment in seeds and endocarp development. The average weight of single seeds, independently of the pollination treatment applied, was 43.8 mg, while the weight of the remaining tissues of the decaying seeds was only 8.7 mg (*p* < 0.0001) (Table 6). On the contrary, once again, the total weight of seeds in endocarps containing two healthy seeds was significantly higher (*p* < 0.0001) than the weight of single seeds, but the average weight of each twin seed was reduced when compared to the weight of average single seed (Table 6). The effect of the seed number per fruit was again largely attenuated on the weight of the endocarp (maternal tissue only) that was heavier as the number of seeds enclosed increased (27.7, 29.5, 38.0 and 42.3 mg for stones containing one dead seed, and one, two or three healthy seeds, respectively). As before, the variability found in the weight of late-aborted seeds was higher than the variability found in the weight of single and double seeds. The coefficient of variation of the weight of the remnants of late-aborted seeds (39.1%) was more than three times higher than the coefficient of variation found in single-seeded fruit (12.8%) (Table 6). Size variability in stone size depending on the number of seeds enclosed was lower.

In ‘Sikitita’, ten of the fruits were initially bi-seeded, although in two of them the second seed aborted, and in three of them the father of the seeds was the same: ‘Arbosana’. Consequently, paternity could be assigned to 77 seeds of fruit from open-pollinated flowers, and in 42 of them the seed was produced by pollen of ‘Arbosana’, 13 seeds were fathered by ‘Tosca’ and 11 by ‘Arbequina’ and the same by ‘Koroneiki’ (Table 2). The analysis of variance shows that ‘Arbosana’ fathered significantly more seeds of ‘Sikitita’ than the other pollinizers (*p* < 0.01).

In five of eight cases in which the paternity of both seeds could be determined, the fathers of the seeds were different, while in three the father was the same: ‘Arbosana’. In addition, ‘Arbosana’ pollen itself also fathered one seed in four of the five remaining bi-seeded fruits, while the father of the second seed was different (either ‘Tosca’ or ‘Arbequina’). The last bi-seeded fruit produced seeds fathered by ‘Arbequina’ and by ‘Koroneiki’ pollen. In addition to these eight bi-seeded fruits, we found two more cases in which the paternity of the second seed could not be established due to the poor quality of the DNA. ‘Tosca’ in one fruit and ‘Arbosana’ in the second case were determined to be the fathers of the surviving seeds.

If, as in the other cultivars, some preferences for a pollinizer were revealed by the analyses, no significant effects of the pollinizers were proved on the seed weight of ‘Sikitita’, which, on average, was remarkably similar for seeds fathered by ‘Arbosana’, ‘Arbequina’ and ‘Koroneiki’. Pollen from ‘Tosca’ again produced the lightest seeds (Table 3). The differences in weight between the seeds fathered by ‘Tosca’ and ‘Arbosana’ (once again the most successful father producing the heaviest seeds) did not reach statistical significance (*p* = 0.06). Within each class, the variability was high for some pollinizers, with seeds weighing little more than 22 mg while other seeds weighed more than 52 mg (Table 6).

As shown in the other two cultivars, the weight of the seed was significantly related to the weight of the fruit (*r* = 0.6; *p* = 0.003), as it was to the weight of the pulp with similar values (*r* = 0.61; *p* = 0.004). In the small sample of this cultivar, four out of twenty fruits were bi-seeded, although in one case both seeds were dead and, in another fruit, one of the seeds aborted. Additionally, three fruits only contained empty endocarps due to seed abortion. Removing them from the analysis greatly improved the prediction of fruit and mesocarp weights (*r* = 0.75 in both cases; *p* = 0.0003 and 0.0004, respectively). The linear equation of fruit weight = 13.02 × seed weight + 1.93 (in grams) described well the significant relationship (*r*^2^ = 0.39; *p* = 0.003) between seed and fruit size, considering all fruit regardless of whether the seed was dead or alive, and of whether it was single or double. The coefficient of regression is enhanced if we only consider single-seeded fruit (*r*^2^ = 0.58; *p* = 0.0004). As observed in ‘Koroneiki’, a closer relationship between endocarp weight (maternal tissue only) and whole fresh fruit weight was found (*r* = 0.68; *p* = 0.0003).

## 3. Discussion

Olive fruits are typically one-seeded drupes, dispersed by birds. Although olive flowers contain four viable equal-sized ovules, distributed two by two in two locules, fertilization is almost always limited to one of the four ovules, because the strong pollen selection in the stigma and style limits the number of pollen tubes accessing the ovary to just one, rarely more [8,9]. This general pattern of seed set has been confirmed here, although deviations were observed, especially in ‘Sikitita’, where two-seeded drupes were not unusual. These results confirm that the frequency of double-seeded fruit is a maternal characteristic. On the other hand, the paternal effect on the seeding pattern was only marginally significant. However, as previously reported in ‘Hojiblanca’ [10], seed abortion in ‘Arbosana’ was more frequent under self-pollination. Similarly, in ‘Koroneiki’, seed abortion was more common under self-pollination, but also under cross-pollination with ‘Arbequina’, a cultivar that seems inter-incompatible with ‘Koroneiki’ [13] (Table 1). The rate of seed abortion in ‘Sikitita’ was not significantly altered by pollination treatments, although it was slightly higher under open-pollination and cross-pollination with ‘Arbequina’, also inter-incompatible with ‘Sikitita’ [5] (Table 1). In short, seed abortion was more likely, but not limited to self-pollination.

Although the differences among pollination treatments in the seeding pattern were only marginally significant, they suggest a trend towards pollen sources potentially affecting seed fate and therefore fruit size. A future line of research is to link seed abortion to the genotype of the pollen parent producing the seed and determine the father in decaying seeds, so as to prove the selective retention of cross-fertilized flowers, as reported in avocado [14]. The significantly higher proportion of fruit with aborted seeds in one of the four ‘Sikitita’ trees used as replicates suggests that nutritional factors might be also involved in the rate of seed abortion. As mentioned, crossings seldom affected the weight of the fruit, although in all cases open-pollination slightly enhanced fruit weight with respect to self-pollination (Table 1). This effect was also observed in some cross-pollination treatments versus self-pollination. However, it is important to note that this slight increase in fruit weight under open- and some cross-pollination treatments was produced even when those pollination treatments were also able to significantly increase fruit set. That is, some pollination treatments produced more and heavier fruit (Table 1), even when fruit weight is known to be negatively affected by fruit load [15,16,17]. Some pollinizers also appeared to more abundantly produce twin seeds in the recipient cultivar. This is the case of ‘Arbosana’ in ‘Sikitita’ fruits. A striking circumstance brought to light for the first time is that twin seeds were very often (7 out of 10 cases) fathered by different pollinizers. Larger differences in seed weight when two different genotypes fathered the twin seeds suggest seed competition within the endocarp, although, as mentioned, abortion of the second seed was seldom observed. In some cases, as in ‘Sikitita’ plants, the second father was ‘Arbequina’, a cultivar inter-incompatible with ‘Sikitita’ [5], suggesting a pollen mentor effect that could be used in breeding. In any case, and despite the low fruit set when ‘Arbequina’ pollen is used in ‘Sikitita’ flowers, the appearance of seeds fathered by ‘Arbequina’ in one-seeded fruits of ‘Sikitita’ (Table 2) proves that incompatibility is not absolute in olive, and that some incompatible pollen grains escape the rejection reaction in the stigma of the recipient flower and achieve fertilization.

Fruit set results confirmed the self-incompatibility of the three evaluated cultivars (Table 1). Although seed paternity did not exactly match the fruit set results, the self-incompatibility of the three cultivars was confirmed, and no single seed was fathered by self-pollen in open-pollinated flowers (Table 2). Analogous results were found by Díaz et al. [18], who found that almost none of the seeds were the product of self-fertilization in ‘Arbequina’ and ‘Picual’ monovarietal orchards of Spain. The same results were observed in ‘Istrska Belica’ in Slovenia [19] and for ‘Barnea’ olives in Israel [20]. Similarly to self-fertilization failure, ‘Arbequina’ pollen failed to produce many seeds in ‘Sikitita’ (Table 2), cultivars previously reported as inter-incompatible [5].

In this experiment, ‘Sikitita’ as a pollinizer was found to be significantly more successful than other cultivars, fathering more seeds in ‘Arbosana’ and ‘Koroneiki’ flowers (not on its own flowers due to its self-incompatibility) (Table 2). ‘Arbosana’, in turn, was an extraordinary pollinizer for ‘Sikitita’ flowers, producing significantly more seeds than other cultivars and promoting high fruit sets in ‘Sikitita’ plants (Table 1 and Table 2). ‘Arbequina’ and ‘Arbosana’, two of the most widely used cultivars for high-density orchards, also seemed to match well (Table 1 and Table 2). In short, some more successful pairs were detected and positive effects of these same pollinizers were found on seed weight (Table 3). In contrast, ‘Koroneiki’ pollen not only failed to father a large number of seeds in ‘Arbosana’ fruits, but it also produced ones with a lighter weight (Table 2 and Table 3). So, a trend toward more and heavier seeds was observed for some successful crosses, and the reverse, fewer and lighter seeds, for less successful crosses.

**Figure 1:**
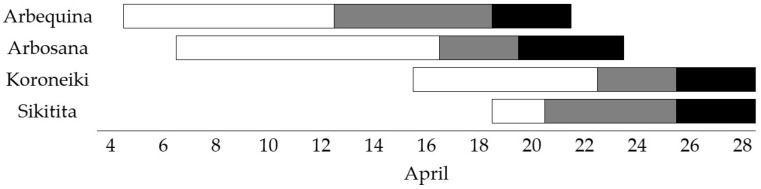
Bloom phenology in the experimental orchard of the four cultivars used in pollination treatments. Season 2023. In white, the start of full bloom; in grey, full bloom and in black, the end of full bloom. Modified from Cuevas et al. [4].

Flowering levels, bloom phenology and proximity to the recipient trees are some key factors that might justify the greater success of some cultivars to father more seeds in inter-compatible varieties. In this experiment, all selected trees were in their “on” year and showed ample bloom overlap (Figure 1). The reduced distance between tree rows of the cultivars here assessed rules out effective pollination distance (30–40 m) [21,22] as a key factor, although the proximity of ‘Sikitita’ to ‘Arbosana’ and ‘Koroneiki’ plants in adjacent rows may favor the high paternity success of ‘Sikitita’. Several factors influence the choice of pollinizers in olive. First, as already discussed, the main cultivar and pollinizer must be inter-compatible; second, bloom dates must overlap; and third, pollen grains from the pollinizer should be abundant and highly viable. Choosing non-alternate bearing cultivars as pollinizers is also advisable, as they are sharing the same fruit destination (olive oil versus table olives). In this work, we have determined the compatibility relationships between different olive oil cultivars widely used for high-density orchards and have observed enough overlaps in bloom dates. Although the flowering dates of ‘Tosca’ were not monitored, because it was not included as a pollination treatment, the work of Maldera et al. [23] confirms that ‘Tosca’ has a prolonged blooming that completely overlaps the bloom dates of ‘Arbequina’, ‘Arbosana’ and ‘Koroneiki’ during the three seasons under study. Although Maldera et al. [23] experiments were carried out in southern Italy, the order of flowering between cultivars is rarely modified by location and year [24,25].

The number of seeds enclosed in the endocarp affected seed and endocarp development. Fruit with aborted seeds had smaller stones, while the stones that enclosed two seeds were heavier. It is worth mentioning that the weight of decaying seeds was highly variable in all cultivars, with values sometimes close to that of viable seeds, while in some cases the aborted seeds were very light (Table 4, Table 5 and Table 6). This variability is interpreted as indicative of seed abortion taking place at different moments. Bradley and Griggs [9] reported common seed abortion in olive in the pro-embryo stage. Early seed abortion almost inevitably leads to fruit drop in olive, but late seed abortion allows fruit to reach harvest, although it reduces fruit weight, as observed in other drupes such as cherry and peach [26]. Our results suggest that the later the abortion, the smaller the weight loss. Seed abortion after the hardening of the stone (July in the Northern Hemisphere) may hardly influence endocarp weight, although pulp weight can still be reduced. Contrarily to empty endocarps, the stones enclosing two seeds were significantly heavier than the stones containing only one seed. Previous works also reported the formation of bi-seeded fruits in ‘Hojiblanca’, which are heavier than fruit containing one seed [10]. The same conclusions were reached in a similar experiment conducted by Farinelli et al. [27], who found maternal effects on the seeding pattern of the cultivars ‘Carolea’ and ‘Kalamon’, the former producing more bi-seeded fruits. Bi-seeded fruits were heavier in ‘Carolea’, but not in ‘Kalamon’.

It is important to underline, however, that sharing fruit with a twin seed was negative for both seeds. In this regard, single seeds were heavier than each seed from the endocarps containing two seeds (Table 4, Table 5 and Table 6). Therefore, double seeding is detrimental to seed weight. In the case of drupes, the negative effects of forming twin seeds are not limited to the size of the seed, but also, and especially, to the growth of the seedlings and their survival. As the dispersion unit in olive and other drupes is the endocarp, the germination and growth of the twin seeds in the same spot after bird dispersal would likely negatively affect seedling growth and survival in nature. However, it is worthwhile mentioning that the abortion of the second seed rarely occurred and very often both seeds seemed healthy, and, although smaller, they had similar sizes, suggesting that sibling rivalry within the endocarp is not critically compromising seed development. Although the single seeds were heavier than each of the twin seeds were, the summation of the weight of the twin seeds is higher than the weight of any single seed, and the endocarps containing two seeds tended to be heavier as it was the fruit containing them. Therefore, despite the negative effect on size observed in twin seeds, maternal investment in these fruits was higher, resembling the well-known effect of seed number on fruit size in berries, pomes, and compound fruits [28,29,30,31,32]. This relationship between seed number and fruit size is, in fact, a fundamental aspect of reproductive biology that affects crop productivity in many species [33,34].

In addition, a significant relationship was found between the weight of single seeds and the stone enclosing them, while the second sampling also demonstrated a significant relationship between seed and fruit weights, and, more importantly, with pulp weight (seeds and stones contribute a little less than 5% to the oil production of the olive fruit [35]). This suggests that genotypes and crosses leading to the production of larger seeds may increase fruit size, yield and olive oil production. These relationships need to be confirmed with a larger sample, however. The relationships between seed and fruit weight were tighter if we remove from the analyses aborted and twin seeds that have a distinct effect on fruit growth (Supplementary Table S1).

Finally, single-seeded fruit was confirmed as the norm in all olive cultivars. This seed set pattern is based on the intense pollen tube attrition performed by maternal plants in the stigma and transmitting tissue of the style, so that only one tube is granted access to the ovary in olive. Such a strong selection of the genotype of the pollen grain that achieves fertilization must have positive effects on sibling performance. In this regard, the extensive overlap of genetic expression between the gametophyte (growing pollen grain) and the sporophyte [36,37] makes it possible to think that such a strong pollen tube selection occurs for the benefit of the siblings in terms of seed germination and seedling growth [38]. A clear relationship between seed size and seedling establishment and survival has been frequently demonstrated in many species in the past ([39,40] and references therein). The same effects are expected to be had on olive seedlings in nature.

## 4. Materials and Methods

### 4.1. Location, Plant Material and Management

**Figure 2:**
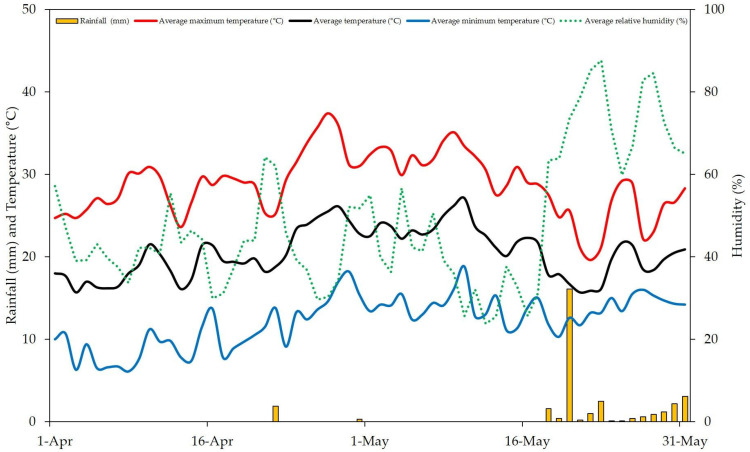
Maximum, minimum and average temperature (in solid lines), and average relative humidity (dashed lines) as well as rainfall (in bars) during bloom and initial fruit set in season 2023 in the experimental orchard. Modified from Cuevas et al. [4].

**Figure 3:**
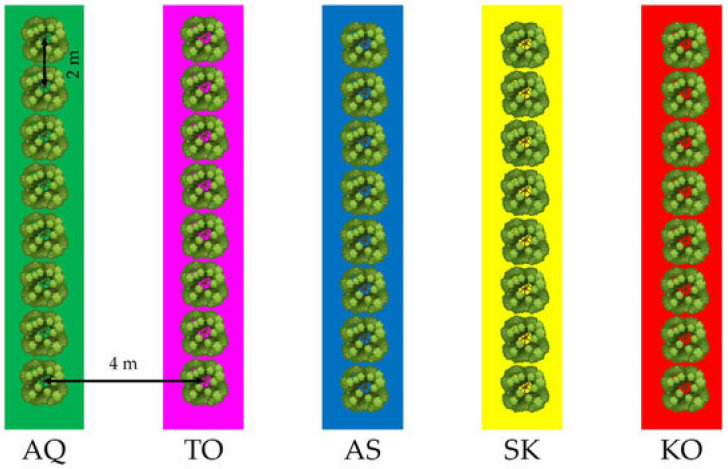
Experimental orchard layout. In green, ‘Arbequina’ (AQ) tree row; in pink, ‘Tosca’ (TO) (syn. ‘Urano’) tree row; in blue, ‘Arbosana’ (AS) tree row; in yellow, ‘Sikitita’ (SK) tree row and in red, ‘Koroneiki’ (KO) tree row. Reproduced from Cuevas et al. [4].

Pollination experiments aiming to characterize the self-incompatibility behavior of three low-vigor olive cultivars, ‘Arbosana’, ‘Sikitita’ and ‘Koroneiki’, were carried out in 2023 in a high-density orchard located on the Rabanales Campus of the University of Córdoba (Córdoba, Spain; 37°56′05″ N, 4°43′00″ W, at 160 m altitude). The experimental area has a subtropical Mediterranean climate according to the classification made by Papadakis [41], with warm dry summers and mild wet winters. The average annual temperature is 18.4 °C (with the average maximum temperature being 25.5 °C, while the average minimum temperature is 11.4 °C). Mean annual rainfall in the experimental site is around 570 mm and the average relative humidity ranges between 40 and 80%. The hours of sunlight reach an average value of 2903 h per year. Weather conditions during bloom and fruit set are shown in Figure 2. The trees used for the experiments were planted in 2011, spaced at 4 × 2 m and trained as an N–S-oriented hedgerow in a multivarietal orchard. The orchard layout showing the tree row arrangement of the different cultivars is depicted in Figure 3. The trees were drip-irrigated with an annual volume of 1000 m^3^ ha^−1^. Pests and diseases were controlled by IPM programs.

### 4.2. Pollination Treatments and Experimental Design

‘Arbosana’, ‘Koroneiki’ and ‘Sikitita’ response to cross-pollination was tested using four trees per cultivar as replicates. Different pollination treatments were applied to each cultivar. Self-, open-, and reciprocal cross-pollination treatments, as well as cross-pollination with a fourth cultivar, ‘Arbequina’, were compared. Each pollination treatment was applied to eight 1-year-old shoots per tree (32 per pollination treatment in total in the four trees), distributed around the tree canopy at the observer’s height. Each tagged shoot bore 10 inflorescences; this uniform flowering level was established by removing excess inflorescences from the shoot.

The treatment of self-pollination was achieved by individually bagging each shoot with hand-made tissue paper bags, before blooming. Cross-pollination treatments were performed by applying, with a camel paintbrush, fresh pollen collected from nearby trees of the selected pollen donor cultivars to the stigmas of the open flowers of each variety (pollen recipient). Cross-pollination was performed on freshly open flowers every other day and at least three times during the bloom period. After hand cross-pollination, the cross-pollinated shoots were re-bagged to minimize pollen access from undesired olive cultivars. Pollination treatments were applied repeatedly starting when the first flowers of the tagged shoots opened and ending when the last flowers wilted. Pollination began on 13 April and ended on 22 April. Open-pollinated flowers were left unbagged and continuously exposed to pollen arrival from different sources during the whole bloom period. Open-pollination in a multivarietal orchard, as in the experiment, represents the optimal pollination treatment due to the unrestricted and continuous pollen arrival from different sources and from different pollen donor trees. None of the flowers were emasculated, emulating natural conditions, so the flowers might receive self-pollen in addition to the corresponding cross-pollination treatment.

The experimental design to determine each cultivar response to pollination treatments was a split-plot where the four trees acted as blocks, each one containing all pollination treatments (self-, open- and cross-pollinations) performed on subsamples represented by the eight 1-year shoots. In those shoots, final fruit set was calculated as the number of fruits per inflorescence (since the inflorescence is the unit of fructification in olive) 45 days after pollination, once fruitlet competition for resources has ceased and the fruit population is firmly established. Fruit weight at harvest was determined for each pollination treatment in each cultivar using a digital scale (Model EK1200i, A&D, Seoul, Republic of Korea) with a precision of 0.001 g.

**Figure 4:**
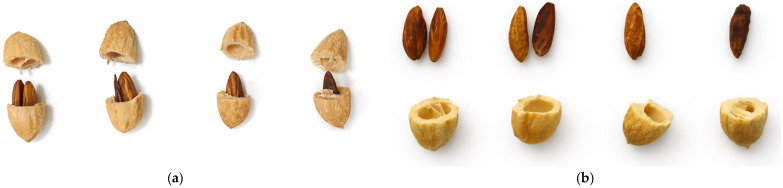
Endocarps and seeds of ‘Koroneiki’ fruits. Seeds within the endocarps (**a**); seeds extracted from the corresponding endocarp (**b**). From right to left, cracked stones containing one aborted seed, one healthy seed, two seeds, one of them dead, and two healthy twin seeds (not always from the same locule).

Finally, samples of 20 fruits per pollination treatment, around 80 in the case of open-pollination, were randomly selected from tagged shoots of all experimental trees at harvest and had their pulp removed, leaving the endocarps clean. After drying for 3 days at ambient temperature, the endocarps were individually weighed and cracked using a plumber vice. Once the endocarps were open, we recorded the number of seeds enclosed (zero, when late seed abortion occurred, or one or two), and weighed them, individually when two seeds were present (Figure 4). Then, we recorded the number of fruits that had aborted seeds, healthy single seeds and two healthy seeds for each pollination treatment.

### 4.3. Seed Paternity Analyses

Once the seeds were extracted from the endocarp, their embryo was dissected and used for paternity analyses. Seed paternity analyses were limited here to open-pollination treatment given the uncertainty regarding the pollen parent in this treatment and the similar probability of all cultivars present in the orchard becoming the male parent of the seeds. In this regard, the pollination design of the orchard with adjacent tree rows of each cultivar allows all pollinizers to have similar chances to father the seeds given the huge production of pollen and the long-distance wind-pollination operating in olive [7]. Bloom phenology of the four cultivars included as pollen recipients and pollen donors in the pollination treatments is shown in Figure 1. After the embryos of the seeds were extracted, each embryo was identified, so the correspondence between the genotype of the pollen grains fathering the seed and the weight of the seed could be scrutinized. To allow paternity assessment, reference genotype profiles were obtained from the five different genotypes equally represented in the experimental orchard [‘Arbequina’, ‘Arbosana’, ‘Koroneiki’, ‘Sikitita’ and also ‘Tosca’ (syn. ‘Urano’)], that can act as potential pollen donors. Genomic DNA was extracted from seed embryos and from fresh olive leaves of the reference cultivars using a NucleoSpin™ Plant II kit (Macherey-Nagel, GmbH & Co., Düren, Germany). Seed paternity analyses were carried out in the laboratories of the Faculty of Mathematics, Natural Sciences, and Information Technologies of the University of Primorska (Koper, Slovenia) following the procedure described by Seifi et al. [42], slightly modified. Seed embryos and potential pollen donors were genotyped at eight polymorphic SSR loci, and paternity analysis was carried out using CERVUS 3.0 software, following a previously published protocol [4,5]. Seed paternity could only be attributed to healthy seeds. Therefore, the decaying and aborted seeds were not processed, and their pollen parent was not determined.

Lastly, to confirm seed and fruit size relationships, an additional set of fruits (20 per cultivar) was collected at harvest in the experimental orchard at random from open-pollinated flowers from non-tagged shoots. The fruits were taken to the lab and the fresh weights of the fruit, pulp, endocarp, and seeds included (healthy or aborted) were individually compared.

### 4.4. Statistical Analysis

Self-incompatibility response of each cultivar estimated by their final fruit set under each pollination treatment was examined by analyses of variance (ANOVA). Fruit weight in response to pollination treatments was also compared by ANOVAs, and the treatment means were compared using Tukey’s test (*p* < 0.05). Correlation and regression analyses between seed, endocarp (maternal tissue only) and fruit weights were performed, while Chi-square analyses were used to check deviations of the seeding pattern in each cultivar under different pollination treatments, among different recipient cultivars and among the replicates of each cultivar to detect seeding pattern variation depending on tree location in the row. The individual and total weights of the seeds included within the endocarp (one, two or aborted) were compared by ANOVA for each recipient (maternal) cultivar, using the four experimental trees as replicates, with the means separated using Tukey’s test (*p* < 0.05) when statistical differences were reached. Finally, the effects of the different male parents on the weight of the seeds fathered by them in open-pollinated flowers were compared two-by-two by Student’s *t*-tests, since the number of seeds for each father (parental genotype) was different. All statistical analyses were performed with Statistix 8.0 (Analytical Software, Tallahassee, FL, USA).

## 5. Conclusions

The results confirm the self-incompatibility of the three olive cultivars assessed. None of the seeds from open-pollinated flowers were the result of self-fertilization, ratifying the preferential allogamy in this species. Fruit set results confirm the existence of inter-incompatibility between some cultivars, namely ‘Koroneiki’ and ‘Sikitita’ with ‘Arbequina’ as the pollen donor. Seed paternity analyses also suggest the existence of some more successful paired crosses, with some pollen donors, fathering a higher number of seeds. A striking discovery brought by seed paternal analyses is that the fathers of the twin seeds formed in a single fruit were often the result of fertilization by different fathers.

Maternal effects on seeding patterns were confirmed, while paternal effects were only marginally significant. Nonetheless, seed abortion was more common under self-pollination and twin seeds were more common in some cross-pollination treatments. Pollinizers rarely influenced seed weight. In contrast, the number and size of healthy seeds within the endocarp significantly affected fruit development. Total seed and endocarp mass increased as the number of viable seeds did. Seed, endocarp, pulp, and whole fruit fresh weights were positively correlated, indicating that larger seeds reclaimed larger endocarps to accommodate them and produced heavier fruit and more olive oil.

## Figures and Tables

**Table 1:** Final fruit set (fruit per inflorescence) 45 days after bloom and fruit weight (g) at harvest in ‘Arbosana’, ‘Koroneiki’ and ‘Sikitita’ in response to different pollination treatments (in the first column).

Pollination Treatment	Final Fruit Set			Fruit Weight (g)		
	‘Arbosana’	‘Koroneiki’	‘Sikitita’	‘Arbosana’	‘Koroneiki’	‘Sikitita’
Self-	0.32 b	0.24 c	0.11 b	1.22 a	0.90 a	1.97 ab
Open-	1.23 a	1.13 a	0.61 a	1.28 a	1.04 a	2.12 ab
× Arbequina	1.06 a	0.26 c	0.25 b	1.21 a	0.92 a	1.88 b
CP1 *	1.25 a	0.68 b	0.50 a	1.22 a	0.89 a	1.99 ab
CP2 *	1.13 a	0.79 ab	0.50 a	1.26 a	0.97 a	2.20 a
*p*	<0.01	<0.01	<0.01	0.71	0.36	0.02

* CP: reciprocal cross-pollination treatments. In ‘Arbosana’, CP1: × ‘Sikitita’ and CP2: × ‘Koroneiki’. In ‘Koroneiki’, CP1: × ‘Sikitita’ and CP2: × ‘Arbosana’. Finally, in ‘Sikitita’, CP1: × ‘Arbosana’ and CP2: × ‘Koroneiki’. Within columns, means followed by different letters differ significantly following ANOVA with a significance level of *p* < 0.05; Tukey’s test was used to separate the means.

**Table 2:** Number of seeds fathered by the different genotypes in ‘Arbosana’, ‘Koroneiki’ and ‘Sikitita’ fruits in open-pollinated flowers according to the paternity analyses.

Recipient Cultivar	Pollen Donors				
	× ‘Arbosana’	× ‘Koroneiki’	× ‘Sikitita’	× ‘Arbequina’	× ‘Tosca’
‘Arbosana’	0	5	**37**	**27**	7
‘Koroneiki’	16	0	**41**	9	6
‘Sikitita’	**42**	11	0	11	13

In bold, more successful pollinizers according to the number of seeds fathered.

**Table 3:** Healthy single seed weight (mean ± SE) (in milligrams) obtained at harvest in ‘Arbosana’, ‘Koroneiki’ and ‘Sikitita’ trees fathered by different pollen donors in open-pollination flowers. Paternity confirmed by the analysis of the DNA of the embryos.

Recipient Cultivar	Pollen Donors				
	× ‘Arbosana’	× ‘Koroneiki’	× ‘Sikitita’	× ‘Arbequina’	× ‘Tosca’
‘Arbosana’	– *	36.5 ± 1.4	**41.2 ± 1.1**	39.4 ± 1.3	39.0 ± 2.8
‘Koroneiki’	**34.7 ± 1.2**	–	34.0 ± 0.8	**34.7 ± 1.2**	33.5 ± 1.4
‘Sikitita’	**43.1 ± 0.9**	42.1 ± 1.2	–	**43.0 ± 1.1**	39.1 ± 1.9

* No seeds produced from self-fertilization were found in any recipient cultivar. In bold, crosses that produced the heaviest seeds.

**Table 4:** Basic statistics of the weight (in milligrams) of ‘Arbosana’ seeds depending on the number of them enclosed in the endocarp.

Viable Seeds per Fruit	Mean	Maximum	Minimum	CV (%)	SD	*n*
0	10.0	22.0	3.5	60.7	6.1	15
1	40.3	64.3	23.1	17.4	7.0	156
2	36.3	46.0	31.6	18.2	6.6	4

**Table 5:** Basic statistics of the weight (in milligrams) of ‘Koroneiki’ seeds depending on the number of them enclosed in the endocarp.

Viable Seeds per Fruit	Mean	Maximum	Minimum	CV (%)	SD	*n*
0	7.9	23.4	2.7	61.4	4.9	14
1	33.6	45.9	18.6	14.2	4.8	148
2	23.0	31.2	12.0	34.8	8.0	4

**Table 6:** Basic statistics of the weight (in milligrams) of ‘Sikitita’ seeds depending on the number of them enclosed in the endocarp.

Viable Seeds per Fruit	Mean	Maximum	Minimum	CV (%)	SD	*n*
0	8.7	12.0	4.0	39.1	3.4	14
1	43.8	58.8	25.9	12.8	5.6	154
2	76.1	93.4	41.7	21.1	16.1	17

## Data Availability

Data are contained within the article and Supplementary Materials.

## Supplementary Materials

The following supporting information can be downloaded at: https://www.mdpi.com/article/10.3390/plants15050813/s1, Table S1. Equations defining the relationships of seed and fruit sizes in one-seeded fruits with healthy seeds in ‘Arbosana’, ‘Koroneiki’ and ‘Sikitita’ samples.
