# Supplementary material for: Pollinizer’ Effects on Olive Seed Set, Size and Abortion
Source: Plants (Basel). 2026 Mar 6;15(5):813. doi: 10.3390/plants15050813 (PMC12986728; doi:10.3390/plants15050813)
Supplement: Supplementary file 1 [file plants-15-00813-s001.zip › plants-4177784-supplementary.pdf]

Table S1. Equations defining the relationships of seed and fruit sizes in one-seeded fruits with healthy seeds in 'Arbosana', 'Koroneiki' and 'Sikitita' samples.

Cultivar	Equation	R^2 value	P value	*n*
'Arbosana'	Fruit weight = 10.22 × seed weight + 1.00 (in g)	0.43	0.0061	16
'Koroneiki'	Fruit weight = 16.48 × seed weight + 0.37 (in g)	0.54	0.0018	15
'Sikitita'	Fruit weight = 19.54 × seed weight + 1.58 (in g)	0.34	0.0022	15
